# miRNA-Based Regulation of Alternative RNA Splicing in Metazoans

**DOI:** 10.3390/ijms222111618

**Published:** 2021-10-27

**Authors:** Anna L. Schorr, Marco Mangone

**Affiliations:** 1 Molecular and Cellular Biology Graduate Program, School of Life Sciences, 427 East Tyler Mall, Tempe, AZ 85287, USA; aschorr@asu.edu; 2 Virginia G. Piper Center for Personalized Diagnostics, The Biodesign Institute at Arizona State University, 1001 S McAllister Ave., Tempe, AZ 85287, USA

**Keywords:** microRNA, alternative splicing, cancer, tissue differentiation, SR proteins, hnRNPs, PTBP1, PTBP2, RBFOX, Quaking, CELF, *C. elegans*

## Abstract

Alternative RNA splicing is an important regulatory process used by genes to increase their diversity. This process is mainly executed by specific classes of RNA binding proteins that act in a dosage-dependent manner to include or exclude selected exons in the final transcripts. While these processes are tightly regulated in cells and tissues, little is known on how the dosage of these factors is achieved and maintained. Several recent studies have suggested that alternative RNA splicing may be in part modulated by microRNAs (miRNAs), which are short, non-coding RNAs (~22 nt in length) that inhibit translation of specific mRNA transcripts. As evidenced in tissues and in diseases, such as cancer and neurological disorders, the dysregulation of miRNA pathways disrupts downstream alternative RNA splicing events by altering the dosage of splicing factors involved in RNA splicing. This attractive model suggests that miRNAs can not only influence the dosage of gene expression at the post-transcriptional level but also indirectly interfere in pre-mRNA splicing at the co-transcriptional level. The purpose of this review is to compile and analyze recent studies on miRNAs modulating alternative RNA splicing factors, and how these events contribute to transcript rearrangements in tissue development and disease.

## 1. Introduction

Multicellular organisms possess widely conserved post-transcriptional regulatory mechanisms used to develop and maintain cell and tissue identities. One of these mechanisms is alternative RNA splicing, which leads to the production of multiple protein isoforms from individual pre-mRNA molecules. Alternative RNA splicing is conserved in multicellular eukaryotes and occurs more frequently in complex organisms [1]. An estimated 95% of human multi-exonic genes are alternatively spliced [2].

Alternative RNA splicing is largely controlled by two well-characterized families of RNA binding proteins (RBPs): serine/arginine (SR)-rich proteins and heterogeneous nuclear ribonucleoproteins (hnRNPs). These two classes of proteins act in a dosage-dependent manner, competing for binding to specific sequences in pre-mRNAs to promote or inhibit exon inclusion, respectively [1]. SRs and hnRNPs are essential for constitutive as well as alternative RNA splicing. In most cases, SR proteins promote mRNA splicing activity and exon inclusion by binding to exonic (ESE) and intronic (ISE) splicing enhancer sequences on pre-mRNA molecules and recruit components of the spliceosome to the respective 5′ and 3′ splice sites [3,4,5]. However, in specific situations, SR proteins can also promote alternative exon skipping [2]. SR protein activity is restricted in regulating constitutive and alternative RNA splicing. Many of them have additional roles in modulating mRNA nuclear export, nonsense-mediated decay (NMD), and protein translation enhancement [6].

hnRNPs are instead typically described as inhibitors of splicing activity and exon inclusion. They bind to exon splicing silencers (ESSs) and intronic splicing silencers (ISSs) and prevent SR proteins and the spliceosome from binding to the pre-mRNA molecule [4,5]. However, similarly to SR proteins, the hnRNP family has highly diverse functions, which involve mRNA maturation, stabilizing mRNA during cellular transport, and activation or silencing of mRNA translation. hnRNPs can also promote or inhibit RNA splicing activity in specific circumstances [5,7,8].

While the SR and hnRNP proteins have been extensively studied and are fairly well characterized, numerous other RBPs are essential for the diversity seen in alternative RNA splicing. Examples of these RBPs include the RBFOX, Quaking, CUGBP and ETR-3-like factor (CELF), and RNA binding motif (RBM) protein families [9,10].

**Figure 1:**
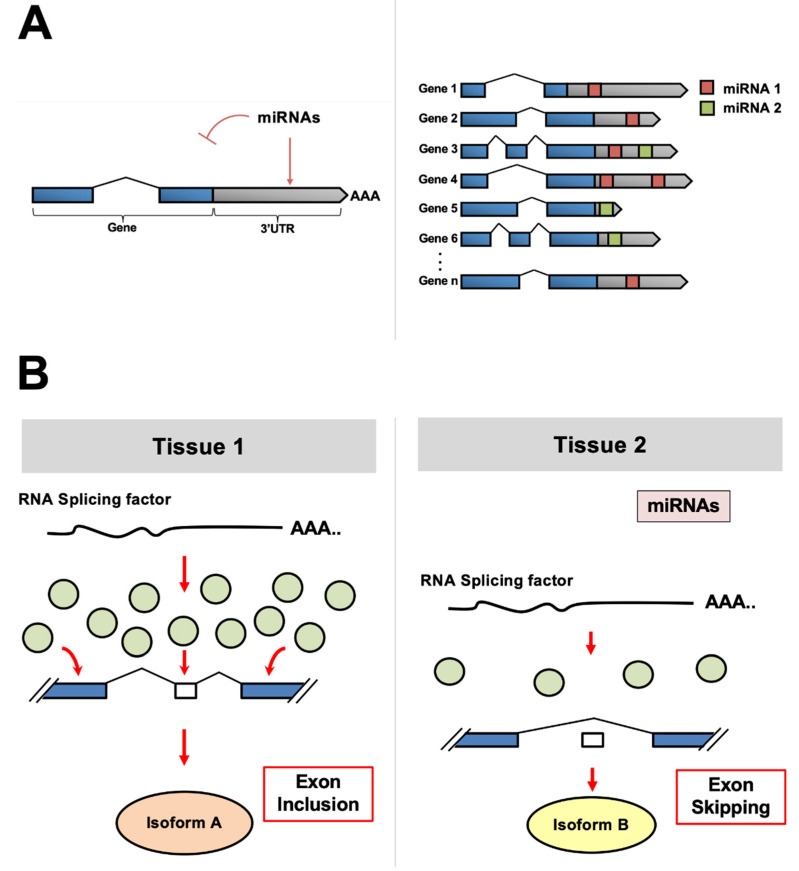
(**A**) miRNAs bind to target genes within their 3′UTR and repress their translation (**Left**). Since the pairing between the miRNAs and its target sequence is not perfectly complementary, a single miRNA may target and repress multiple mRNA transcripts (**Right**). (**B**) As shown in Tissue A and Tissue B, miRNAs may differentially regulate the abundance of RNA splicing factor proteins (shown as light green circles). Tissue 1 (**Left**) has a higher dosage of the RNA splicing factor due to a lack of miRNAs available to inhibit their translation. In this specific example, the abundance of splicing factors leads to an intron inclusion event (Isoform A). In contrast, in Tissue 2 (**Right**), miRNAs that target the RNA splicing factor lower its abundance, interfering with the splicing pattern of target genes. In this specific example, this leads to an intron exclusion event (Isoform B).

Despite the degree of specificity that can be achieved through several RNA splicing factors, other post-transcriptional regulatory mechanisms allow fine-tuning for tissue development and differentiation. Specifically, microRNAs (miRNAs), which are short non-coding (~22 nt) RNA molecules, exert a considerable influence by targeting motifs within the 3′ untranslated region (3′UTR) of genes, leading to inhibition of translation of target mRNAs [11] (Figure 1A). Although transcribing then degrading mRNA may seem counterproductive, it has been hypothesized that this mechanism evolved in metazoans to provide a way to “sculpt the transcriptome” to achieve optimal gene expression in various tissues after transcription occurs [11].

In metazoans, mature miRNAs are incorporated into the microRNA-induced silencing complex (miRISC), which recruits deadenylase complexes to the target 3′UTRs [11]. This pairing has been shown to require as little as six consecutive nucleotides in the 5′ end, or seed region, of the mature miRNA. Perfect complementarity within the seed region is considered the canonical indicator of miRNA targeting. However, many recent studies indicate that miRNAs can target non-canonical elements in target mRNAs [12,13]. Currently, bioinformatic approaches are the only tools for high-throughput miRNA target detection. Based on these algorithms, it is proposed that each miRNA controls hundreds of gene products [14] (Figure 1B).

The human genome contains ~2000 distinct mature miRNAs located in intergenic regions, introns, and operon-like clusters. Metazoan miRNAs were first characterized in *C. elegans* as heterochronic regulators that temporally repress genes involved in developmental transitions [15,16]. miRNAs and their regulatory mechanisms are conserved from humans [17] to Cnidarians [18]. In the past years, several groups produced tissue-specific localization data for many miRNAs in worm, mouse, rat, and human somatic tissues [19,20,21] and in cancers [22]. These results unequivocally show distinct functional miRNA populations in tissues, which can reshape transcriptomes and contribute to cell identity acquisition and maintenance.

In recent years, several intriguing studies connected miRNA targeting to alternative RNA splicing. This novel form of regulation is achieved indirectly through miRNA targeting and lowering the dosage of several RNA splicing factors, which in turn change the splicing pattern of many genes in a tissue-specific manner. The degree of regulation that miRNA pathways exert in alternative RNA splicing and tissue differentiation has yet to be fully understood at an organismal level, but it is exciting. In this view, miRNAs do not only regulate gene expression at the post-transcriptional level, as canonically described, but also indirectly at the co-transcriptional level, throughout the modulation of these RNA splicing factors. In *C. elegans*, for example, virtually all SR and hnRNP orthologs are targeted by miRNAs, and the depletion of the miRISC leads to widespread changes in splice junction usage [19]. These broad splicing errors have also been observed in transcriptomes from Dicer knockout mice [23], suggesting that this form of gene regulation is not only present in vertebrates but may have evolved earlier in the tree of life.

In humans, it is very common for RNA splicing factors to contain predicted or validated miRNA targets (TargetScan) in their 3′UTRs. Still, the contribution of miRNAs to protein isoform production has not been fully characterized yet. This review highlights and discusses this new form of miRNA-based regulation and its potential implications in development and disease. Table 1 summarizes the RNA splicing factors with their respective miRNAs discussed below.

## 2. RNA Splicing Factors Regulated by miRNAs

### 2.1. SR Proteins

SR proteins are regulators of both constitutive and alternative RNA splicing, and they are essential for proper tissue development and differentiation. The human genome contains at least 14 SR protein genes [73]. Dysregulation of many SR proteins has been characterized in several types of diseases, especially cancer and developmental disorders, where widespread aberrant RNA splicing events have been observed. As detected in several types of cancers, individual SR proteins may act as oncogenes or tumor suppressor genes, which is dependent on their tissue-specific expression [74,75]. Many SR proteins have miRNA targets in their 3′UTRs and have shown to be targeted and repressed by these miRNAs, leading to widespread RNA splicing errors. Several examples of miRNA-based SR protein regulation have been described for several specific SR proteins and are discussed below.

#### 2.1.1. SRSF1 (SF2/ASF)

**Figure 2:**
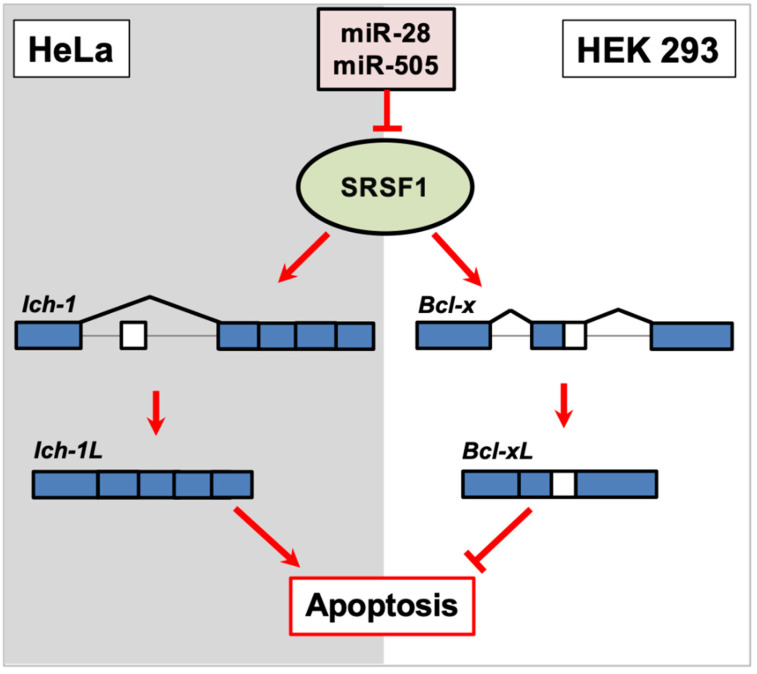
In HeLa cells, SRSF1 promotes exon skipping in *Ich-1* to produce the pro-apoptotic *Ich-1L* isoform. However, in HEK 293 cells, SRSF1 promotes the expression of *Bcl-xL*, the anti-apoptotic isoform of *Bcl-x*, by favoring a distal 5′ splice site. miR-28 and miR-505 directly target the 3′UTR of *SRSF1*, and the result induces a shift of the isoform ratios of *Ich-1* and *Bcl-x*.

SRSF1 is a well-characterized general RNA splicing factor involved in both constitutive and alternative RNA splicing and influences the location of the splice site in a dosage-dependent manner. The RNA splicing function of SRSF1 is different depending on the cell subtype. When overexpressed in HeLa cells, SRSF1 promotes exon skipping in *Ich-1*, a member of the caspase family of proteases, to promote the longer pro-apoptotic *Ich-1L* isoform, which uses a more distal stop codon. In contrast, the shorter anti-apoptotic *Ich-1s* isoform retains the 61 bp exon (Figure 2). However, when SRSF1 is overexpressed in HEK 293 cells, the longer anti-apoptotic isoform of *Bcl-xL*, is favored over the shorter pro-apoptotic *Bcl-x(s)* isoform (Figure 2). The 3′UTR of *SRSF1* possesses several miRNA targets, and coincidentally, the miRNAs that target *SRSF1* are downregulated in many cancers. Verduci et al. demonstrated that the overexpression of miR-28 and miR-505 in mouse embryonic fibroblasts repressed SRSF1, which allowed for the ratios of *Ich-1* and *Bcl-x* isoforms to become more balanced [24]. A previous study also supports these findings [76]. Taken together, these results suggest that miRNA-based regulation is crucial for the precise dosage of RNA splicing factors to produce the correct ratios of alternatively spliced isoforms [24] (Figure 2). However, it is worth noting that overexpressing miR-505 alone repressed SRSF1 expression in mouse embryonic fibroblasts but unexpectedly promoted the anti-apoptotic *Ich-1s* isoform [24], suggesting that other mechanisms are responsible for the proper expression of pro-apoptotic spliced gene isoforms in this mouse model [24].

The relationship between miRNA regulation on SRSF1 expression and alternative RNA splicing has also been studied in neuroblastoma cells. When these cells are treated with retinoic acid, proliferation is inhibited, and apoptosis is promoted [77]. Further studies identified increased expression of miR-10a and miR-10b in these cells, which both target the 3′UTR of *SRSF1*. As predicted, SRSF1 levels decreased in neuroblastoma cells after retinoic acid treatment, which presumably altered the alternative RNA splicing of other mRNA transcripts. This was demonstrated by a switch to exon 10 inclusion in the *tau* gene [25], a key player in neurological disorders.

Interestingly, SRSF1 may also act as an oncogene in several cancers. When overexpressed, SRSF1 acts as a proto-oncogene by inducing alternative RNA splicing in multiple genes that operate in the apoptotic pathway, promoting cancer establishment or progression [78,79]. Predictably, an increase in SRSF1 expression in cancers is correlated with the appearance of new aberrant RNA isoforms [26] and poor prognosis [78,80].

SRSF1 is also targeted by miR-766-3p, a well-characterized tumor suppressor or inducer in several types of cancers [27,81,82,83], which is often markedly downregulated in cancers, leading to the overexpression of SRSF1 [27,82]. Unfortunately, while very important, the exact RNA splicing errors induced by the overexpression of SRSF1 in the absence of this miRNA are unclear and difficult to pinpoint due to the various roles beyond alternative RNA splicing in which SRSF1 is involved.

#### 2.1.2. SRSF2

SRSF1 is not the only member of the SR protein family known to be targeted by miRNAs. SRSF2, a second member of the SR family of proteins, similarly to SRSF1, is also widely expressed and has a diverse role in many biological processes [84]. This gene is frequently upregulated in many human cancers, and this event is associated with poor prognosis in patients. Similar to SRSF1, SRSF2 overexpression leads to global alternative RNA splicing errors [29,30]. SRSF2 promotes apoptosis through alternative RNA splicing of specific cancer genes, such as *c-flip*, caspases-8 and -9, and *Bcl-x*, to their pro-apoptotic isoforms, and a decrease in its expression is associated with the inhibition of apoptosis [31,32]. Because of the significant role SRSF2 plays in alternative RNA splicing in apoptotic pathways, the dysregulation of miRNAs that target *SRSF2* is a fingerprint in many types of cancers. In renal cell cancer, for example, miR-183-5p and miR-200c-3p are significantly upregulated and directly target *SRSF2* to prevent the activation of apoptotic pathways [28,32,83]. Another miRNA, miR-193a-3p, which is upregulated in multiple other cancers, has been shown to directly target *SRSF2* in nasopharyngeal cancer [33]. Interestingly, *SRSF2* is not the only SR protein gene targeted by miR-193a-3p, as this miRNA has been shown to also directly target *SRSF6* [34]. Unfortunately, the global RNA splicing defects caused by the change in SRSF2 expression levels by miRNAs have not yet been characterized in detail.

#### 2.1.3. SRSF6

SRSF6 is another SR protein member also known to be targeted by miRNAs. SRSF6 is unfortunately less well characterized when compared to other SR proteins, but it has been shown to be dysregulated in a variety of human diseases. SRSF6 overexpression has been observed in lung and colon cancers [85] and Huntington’s disease [35], but surprisingly, it is downregulated in pancreatic cancer [34].

Among other genes, SRSF6 modulates alternative RNA splicing of the genes *OGDHL* (oxoglutarate dehydrogenase-like protein) and *ECM1* (extracellular matrix protein 1), both known to play roles in cancer metastasis. Upregulation of miR-193a-5p (also a regulator of SRSF2 described previously) directly targets and inhibits *SRSF6*, and as a result, the splice variants of *OGDHL* and *ECM1* conducive to tumorigenicity are expressed in pancreatic cancer [34].

In a mouse model studying miRNA-based treatments for Alzheimer’s disease (AD), increased activity of miR-146a caused the depletion of SRSF6 expression [36]. The exact mechanisms involving miRNA regulation and SRSF6 in neurological disorders have yet to be fully understood, but unbalanced SRSF6 expression alters the splicing pattern of several neuronal target mRNAs, including the *tau* gene [86], a well-known player in AD also expressed with aberrant RNA splicing in other neurological diseases.

Furthermore, SRSF6 is a direct target of miR-506-3p, and its overexpression caused by the repression of miR-506-3p was observed in pleural fibrosis, a thickening of the membranes that cover the lungs [37]. Unfortunately, the downstream effects have yet to be directly identified. As discussed, SRSF6 has also been shown to be up- or downregulated in a variety of diseases, and this regulation was often found to be dependent on SRSF6-specific miRNAs.

#### 2.1.4. SRSF7

SRSF7 is an essential splicing regulator, and its aberrant expression has also been implicated in several types of cancers. SRSF7 is known to direct alternative RNA splicing of several genes, including *BRCA1* [87] and *SPP1* [38], which are well-known regulators of tumorigenesis.

The *SRSF7* 3′UTR contains at least seven miRNA targets (TargetScan), an indicator of the need to precisely dose the abundance of this RNA splicing factor in cells. Boguslawska et al. demonstrated specifically in renal cancer that miR-30a-5p, miR-181a-5p, and miR-216b directly target the 3′UTR of *SRSF7* and repress its expression, and in turn, affect the alternative RNA splicing of *SPP1*. Interestingly, miR-216b repression of SRSF7 led to change in the alternative RNA splicing pattern of *SPP1*, favoring the *SPP1-c* isoform, while decreasing the *SPP1-b* isoform, which is instead boosted in the presence of SRSF7 [38].

These three miRNAs are not the only regulators of SRSF7. MiR-188 was also found to target the *SRSF7* 3′UTR in an acute kidney injury model. This interaction led to a decrease in cell viability, potentially induced by the alteration of RNA splicing patterns of anti-apoptotic gene isoforms caused by the lack of SRSF7 [39].

#### 2.1.5. SRSF9

SRSF9 is an important myogenic regulator. Its depletion, together with other RNA splicing factors, is an essential step in the myogenesis of muscle development. Specifically, miR-1 and miR-206 directly target the *SRSF9* 3′UTR to allow for the transition from proliferation to differentiation in myogenesis [40]. In developed tissues, SRSF9 acts as an oncogene as it represses apoptosis through alternative RNA splicing of genes, such as *Bcl-x* [88]. Continued repression of SRSF9 is dependent on miR-1, which is downregulated in several types of cancers, including bladder cancer [41]. In cervical cancer cells, miR-802, which targets the 3′UTR of *SRSF9*, was found to be downregulated while SRSF9 was upregulated. Upon transfection of miR-802 in cervical cancer cells, SRSF9 expression was inhibited, leading to inhibited cell proliferation, cell cycle arrest, and apoptosis [42]. While a few players have been identified [88], the widespread changes in RNA splicing patterns caused by miRNAs’ deregulation of SRSF9 function in various cancers have yet to be identified.

### 2.2. hnRNP Proteins

Working as antagonists to SR proteins, hnRNP proteins typically promote exon exclusion in a dosage-dependent manner. Dysregulation of hnRNPs is described in several cancers and is also characterized in other diseases, such as amyotrophic lateral sclerosis and neurodegenerative disorders [89]. Additionally, the hnRNP family is comprised of about 20 proteins and is the most abundantly expressed group of RNA binding proteins in mammalian cells [89]. Similar to SR proteins, changes in their abundances lead to widespread RNA splicing errors. A few examples of these errors induced by miRNA deregulation are discussed below.

#### 2.2.1. hnRNP A1

hnRNP A1 is one of the main components in the spliceosome, and is essential for constitutive RNA splicing [90] but is also known to modulate alternative RNA splicing in a dosage-dependent manner [89]. hnRNP A1 is ubiquitously expressed, and dysregulation of different miRNAs that target this gene has been observed in diseases or disorders in distinct tissues. For example, a study by Sokół et al. (2018) found *hnRNP A1* to be targeted by miR-135a, miR-1-3p, miR-206, and miR-149-5p in renal cancer cells and other types of cancer [28]; however, in acute myeloid leukemia, hnRNP A1 is overexpressed due to downregulation of miR-451, which directly targets *hnRNP A1* [43].

A recent study showed that in the presence of miR-424 and miR-503, these two miRNAs were able to directly repress hnRNP A1 and potentially alter the RNA splicing pattern of hnRNP A1-regulated genes, but more extensive studies beyond bioinformatics approaches have yet to support this hypothesis [91].

These various examples involving hnRNP A1 suggest that the abundance of this gene is tightly controlled by miRNA networks, and the deregulation of these networks may lead to dramatic rearrangements in RNA splicing patterns in many transcripts controlled by this RNA splicing regulator, leading to disease states.

#### 2.2.2. hnRNP A2

The RNA splicing factor hnRNP A2 is closely related to hnRNP A1 and is hypothesized to have originated from a duplication event [92]. As previously mentioned, several hnRNP members, including both hnRNP A1 and hnRNP A2, alternatively splice the *PKM* gene to exclude exon 9 and include exon 10 to produce the *PKM2* isoform [93,94,95]. A recent study using exon-tiling microarrays showed that knockdown of hnRNP A2 with RNAi promoted at least two exon inclusion and four exon skipping events in the genes *MTA3*, *MAP9*, *EPB41L4A*, *TP53INP2*, *RXFP1*, and *PHF14* [96], and potentially more, suggesting that hnRNP A2 is also able to induce alternative RNA splicing in a large number of genes.

Similar to *hnRNP A1*, the *hnRNP A2* 3′UTR contains several predicted and validated miRNA targets, suggesting that the dosage of this gene is also tightly regulated in cells. *hnRNP A2* is directly targeted by at least three miRNAs. A 2012 study by Sun et al. found that *hnRNP A2* is repressed by miR-124 and miR-340, while *hnRNP A1* is repressed by miR-137 in a luciferase assay [94], which suggests that despite their similarities, *hnRNP A1* and *hnRNP A2* are regulated by different miRNAs.

Although miRNAs are typically repressors of gene expression, the expression of hnRNP A2, as well as the SFPQ protein (another RNA splicing factor), increased when miR-369 was transfected in mouse adipose-derived mesenchymal stem cells [97]. In this study, hnRNP A1 only showed a slight increase when compared to hnRNP A2 and SFPQ. While an exact mechanism was not established, it is important to note that the 3′UTR of *hnRNP A2* but not *hnRNP A1* contained a predicted miR-369 binding site, which seems to be able to stabilize *hnRNP A2* mRNAs [97,98].

#### 2.2.3. Polypyrimidine Tract Binding Proteins

Polypyrimidine tract binding (PTB) proteins are named because of their ability to bind intronic polypyrimidine tracts during the pre-mRNA splicing event influencing pre-mRNA processing [99]. Although their primary role is to modulate pre-mRNA splicing, they have been shown to also play a role in mRNA metabolism and mRNA transport. The PTB proteins have well-characterized roles in the biogenesis and processing of specific miRNAs, which often form regulatory feedback loops and allow for the transition of specialized cells [100,101,102]. PTBP1 and PTBP2 are the two most well-characterized members of the PTB family. PTBP1 is a major repressive regulator of alternative RNA splicing, causing exon skipping in many alternatively spliced precursor mRNAs [5,103]. PTBP1 is highly expressed in most of the tissue types but is repressed in neuronal tissues, allowing for the inclusion of neuron-specific exons in many genes [44]. Both *PTBP1* and *PTBP2* have several predicted (TargetScan) and validated miRNA binding sites in their 3′UTRs. MiRNAs have been shown to exert considerable influence in neuron-specific alternative RNA splicing by targeting *PTBP1* transcripts, which in turn upregulate neuron-specific PTBP2 expression. Coincidentally, several of these miRNAs, including miR-124 and miR-137 [45,104], are highly abundant in neuronal tissues, which supports the idea that an miRNA-based form of regulation is required for the correct tissue-specific expression of PTB isoforms.

Dysregulation of PTBP1-specific miRNAs is implicated in neurological diseases, including AD and brain cancers. Abnormal RNA splicing of the amyloid precursor protein (APP) in the neurons of AD patients was observed with downregulation of miR-124, a direct target of PTBP1, which instead is upregulated [46].

Several non-neuronal-specific miRNAs are also regulators of PTBP1 in various cancers. PTBP1 promotes the expression of the pyruvate kinase M1/2 isoform (*PKM2)* through alternative RNA splicing, which is expressed in proliferating cells [105] and is crucial for establishing the “Warburg effect” in many types of cancers [106]. Overexpression of PTBP1 in colorectal tumors was attributed to the downregulation of muscle-specific miR-1 and miR-133b, which target the *PTBP1* 3′UTR [47]. Additionally, muscle-specific miR-206 directly targets the *PTBP1* 3′UTR [45] and its dysregulation is implicated in sarcomas [107].

PTBP2, also known as nPTB, is the neuron-specific PTB isoform downregulated in differentiated muscle tissue. Similar to PTBP1, the muscle-specific miR-133 targets *PTBP2* transcripts during myoblast differentiation. Changes in miR-133 expression and its regulation of both PTB isoforms resulted in mRNA splicing errors in several genes including those involved in muscle cell differentiation [48]. Dysregulation of miR-132, a brain-specific miRNA that targets the *PTBP2* 3′UTR, has been implicated in several disorders. In glioblastoma cells, miR-132 promotes proliferation and self-renewal potential by inhibiting PTBP2, which is implicated in cell differentiation [49]. Proper expression of PTBP2 in neuronal cells is crucial for alternative RNA splicing of neuronal genes. For example, in the brain, the tau proteins are equally expressed as two isoforms: 4R-tau and 3R-tau, which either include or exclude exon 10 from the primary *tau* mRNA. PTBP2 was shown to promote the 4R-tau isoform. The upregulation of miR-132, which targets the 3′UTR of *PTBP2*, causes a decrease in PTBP2 expression, leading to a tau isoform imbalance, which is observed in progressive supranuclear palsy [50].

### 2.3. Other RNA Splicing Factors

Several other RNA splicing factor protein families are also essential for alternative RNA splicing and are modulated through miRNA regulation. We will now focus on five of the most well-characterized families: RBFOX, CELF, NOVA, Quaking, and RBM protein families.

#### 2.3.1. RBFOX

The RBFOX protein family is highly conserved across metazoans and consists of three paralogs in mammals: RFBOX1, RBFOX2, and RBFOX3, which are expressed in a tissue-specific pattern [108]. All RBFOX paralogs have been found to synergize or antagonize the activities of other RNA splicing factors, which would allow for a higher level of tissue-specific alternative RNA splicing [108].

The RNA splicing factor RBFOX1 is implicated in mammalian neuronal development, myoblast fusion, and skeletal muscle and heart function [108]. The *C. elegans* RBFOX-1 homolog (*fox-1*), which has been shown to control male or hermaphrodite development by splicing several genes including *xol-1* pre-mRNA [109], is expressed in body muscle tissue and is targeted by miRNAs in intestinal tissue [19]. While the mammalian homologs of RBFOX1 may have distinct or unrelated functions, RBFOX1 is also expressed in muscle tissue and neurons [110], and is needed for proper neuronal development [111], and its absence or alteration has been implicated in a variety of neurological processes and disorders [112]. The *Rbfox1* 3′UTR contains several predicted (PicTar) and validated miRNA targets. A 2016 study identified *Rbfox1* in *Drosophila melanogaster* as a memory-promoting gene and conversely, one of its miRNA partners, miR-980, as a memory suppressor [51]. However, later research clarified that only *Rbfox1* transcripts with extended 3′UTRs were targeted by miR-980 [52], leaving shorter transcripts intact. The ability of genes to express different 3′UTR lengths is possible through the mechanism of alternative polyadenylation (APA). APA has been shown by several groups as a potent mechanism to bypass miRNA regulation [19,113,114,115]. In addition, another miRNA, miR-129-5p, has been shown to directly inhibit the expression of *Rbfox1* and *Rbfox3* transcripts through targeting their 3′UTRs, which both have multiple miR-129-5p binding sites [53].

Another member of the RBFOX family, *Rbfox2*, is directly targeted by let-7g, miR-9, and miR-135a, and is expressed with two tissue-specific isoforms, *Rbfox2_40_* and *Rbfox2_43_*, which differ by an alternatively spliced exon (either 40 or 43 nt long). Although these two isoforms seem to have redundant traits, they are functionally diverse, and their dysregulation results in severe complications [54]. The non-muscle-specific isoform, RBFOX2_40_, is overexpressed in the hearts in myotonic dystrophy type 1 (DM1) patients. A combination of repressed expression of let-7g, miR-9, and miR-135a and elevated expression of the RNA splicing factor CELF1, discussed below, were identified to drive *Rbfox2_40_* expression in a mouse heart model of DM1 by Misra et al. 2020. RNA mis-splicing of *Scn5a* and *Kcnd3* ion channel transcripts and potentially other genes are consequential of abundant *Rbfox2_40_* expression [54], leading to ion channels that function more slowly and are more prone to arrhythmias [116]. Taken together, all these studies indicate the RBFOX family is strongly regulated by miRNAs, which suggest that alternative RNA splicing is also subject to this type of regulation.

#### 2.3.2. CELF Protein Family

CELF (CUGBP and ETR-3-like factor) family protein homologs are widely conserved and are involved in post-transcriptional mechanisms, including RNA editing and translation, in addition to alternative RNA splicing. CELF1 and CELF2 regulate several developmentally related RNA splicing events in the heart and are highly expressed in the brain [23,117]. As previously mentioned, overexpression of CELF1 promotes the expression of the *Rbfox2_40_* isoform observed in DM1 patients [54]. The regulation of CELF1 and CELF2 through miRNAs is essential for proper heart development. Kalsotra et al. (2008) found that the change in CELF1 and CELF2 protein levels decreased by 10- and 18-fold, respectively, from embryonic to adult hearts from mice with no change in mRNA levels. To investigate if these changes in protein levels were regulated by miRNA, Kalsotra et al. (2010) performed a knockout of Dicer in mouse heart tissue, which significantly increased CELF protein expression. Both of the *CELF1* and *CELF2* 3′UTRs contain predicted targets for miRNA-23a/b, which were confirmed by luciferase assays [23]. Furthermore, administration of an miR-23a antagomir in the adult mouse heart sequestered miR-23a/b, which caused a significant increase of CELF1 and CELF2 protein levels and shifted the alternative RNA splicing of pre-mRNAs to embryonic-specific isoforms. In addition to miR-23a/b, luciferase assays indicated that miR-322/-503 also targeted *CELF1* as shown by the luciferase activity decreasing by more than 50% in the presence of these miRNAs with the *CELF1* 3′UTR [61], which suggests CELF1 and CELF2 have distinct patterns of expression or function.

Dysregulation of CELF2 has been described in several cancers, particularly glioblastomas. Profiling of miRNA populations from human glioma tissues and glioblastoma cell lines identified the upregulation of several miRNAs that are predicted to target *CELF2*. Subsequent research utilizing luciferase assays with miR-95-3p and miR-20a provided substantial evidence of their role in CELF2 inhibition, promotion of tumor development, and progression in glioblastomas [61,62,63]. The distinct expression patterns of CELF1 and CELF2 could possibly be reliant on proper miRNA regulation.

#### 2.3.3. NOVA

The NOVA (neuro-oncological ventral antigen) protein family members, NOVA1 and NOVA2, are both RNA splicing factors that were first identified for their roles in neurological diseases. The roles of NOVA1 in cancer are not yet fully understood, but correlations between *Nova1*-targeting miRNAs and NOVA1 expression have been described in multiple types of cancers. *Nova1* is directly targeted by miR-181-5p and miR-203a-3p in neurons [64,65], and is also expressed in various other tissues and is often implicated in cancers. MiR-592 is considerably underexpressed in thyroid cancer while its target, *Nova1*, is overexpressed [66]. NOVA1 is also overexpressed in gastric cancer with the downregulation of miR-339, another miRNA that directly targets the *Nova1* 3′UTR [67]. In addition, miR-146b-5p directly targets *Nova1*, and its upregulation was observed in tissues surrounding the sites of tumors in gastric cancer after gastrectomy [118]. Contradictory to other studies, repression of NOVA1 by the overexpression of miR-27a-3p, which directly targets the *Nova1* 3′UTR, has been identified in gastric cancer [119,120,121]. A study examining the role of miR-7-5p in non-small cell lung cancer (NSCLC) identified *Nova2* as a direct target [68]. In NSCLC cell lines, decreased expression of miR-7-5p and subsequent overexpression of NOVA2 was identified as the cause of tumor cell proliferation, migration, and invasion [68].

#### 2.3.4. Quaking Protein Family

The Quaking (QKI) protein is a member of the STAR (signal transduction and activation of RNA) family of RBPs and was originally named due to the persistent tremors seen in QKI mutant mice [122]. In humans, each of the three QKI isoforms has distinct roles in cell differentiation [55,56,123] despite differing in their C-termini and 3′UTRs [57]. QKI-5 is expressed in the nucleus during embryogenesis and regulates pre-mRNAs for myelination proteins by alternative RNA splicing, and then is downregulated after birth [58]. In contrast, QKI-6 and QKI-7 are expressed later in embryogenesis in the cytoplasm [56], and their mRNAs possess different 3′UTRs than QKI-5 [124]. Several studies indicate that QKI proteins are potent mediators of differentiation in several cell types, for example, neurogenesis in neuronal progenitor cells (NPCs) [124] and endothelial cells that enter endothelial-to-mesenchymal transition (EMT) [125]. EMT is a process where epithelial cells lose cell polarity and adhesion and gain invasive properties typical of mesenchymal cells. EMT is a common hallmark of malignant cancers.

Coincidentally, several miRNAs that specifically target QKI proteins are upregulated during cell proliferation and development. The timing of neuronal differentiation depends on appropriate expression and regulation of miRNAs, such as miR-214-3p, which is upregulated in NPCs during neurogenesis and dendritic development, inhibiting the expression of all three QKI isoforms [59,124]. Similar patterns of QKI inhibition by miR-214-3p have also been identified in epithelial cells during angiogenesis [125]. Additionally, analysis of the 2.3 kb 3′UTR of *Qki-5* has shown multiple predicted binding sites for miR-200c and miR-375; consequently, overexpressing these specific miRNAs significantly decreased QKI-5 expression in epithelial cells, which resulted in a decrease of their target mesenchymal-specific RNA alternatively spliced genes [126]. Additionally, upregulation of miRNA-200c was associated with multiple types of cancers, presumably by decreasing QKI-5 expression, resulting in dysregulation of alternative RNA splicing events [126]. Similarly, in colorectal cancer, *Qki-5* was identified as a target for miR-221, which is a necessary factor for in vivo tumor growth [127]. Furthermore, QKI-5 expression is associated with tumorigenic properties in esophageal squamous cell carcinoma, such as proliferation, tumor invasion, and mitigation. These tumorigenic traits are inhibited, and apoptosis is induced by miR-143-3p, which directly targets *Qki-5* [128]. Similarly, QKI-6 was found to be upregulated in glioblastomas and its targeting miRNA, miR-29a, to be downregulated. The underlying mechanism could be attributed to QKI-6 regulation of WTAP, a protein that stimulates epidermal growth factor (EGF) signaling [60], but more experiments need to be performed to validate this possibility.

#### 2.3.5. RNA Binding Motif (RBM) Proteins

RBM proteins are loosely defined by the presence of at least one RNA-recognition motif (RRM), which they use to target specific mRNAs [129]. Several RBM proteins are involved in cardiomyocyte differentiation, myofibrillogenesis, and muscle-specific alternative RNA splicing.

One of its members, RBM24, is instrumental in cardiac and skeletal muscle development, by promoting exon inclusion to produce muscle-specific protein isoforms of several genes. A recent study by Cardinali et al. (2016) found that the *Rbm24* 3′UTR is directly targeted by miR-222 during the differentiation of skeletal muscle cells. Consequently, the overexpression of miR-222 led to the repression of *Rbm24*, resulting in defective exon inclusion of *Coro6*, *Fxr1*, and *NACA* muscle-specific gene isoforms [70]. This research supports the role of dysregulated miR-222 in several muscular disorders [21].

In addition, another RBM protein, RBM10, is involved in alternative RNA splicing of the conserved gene *Numb*, which possesses both a short and a long gene isoform, *Numb-S* and *Numb-L*, respectively. Expression of *Numb-L* is associated with cell proliferation, and its upregulation leads to various types of cancers [69]. *Rbm10* is targeted by multiple miRNAs, including miR-133a, miR-133b, and miR-335 [69]. Interestingly, in tumors, only miR-335 is increased and inhibits RBM10 expression, leading to the expression of the oncogenic *Numb-L* isoform, specifically in endometrial cancer [69], and perhaps interfering with the splicing pattern of other genes.

Endothelial splicing regulatory proteins 1 and 2 (ESRP1 and ESRP2) are two members of the RBM family that regulate EMT, an event critical for proper tissue differentiation, through their downregulation [130,131]. Dysregulation of EMT events through the overexpression of ESRP1 in particular, has dire consequences for cell differentiation and consequently is typically observed in many types of cancers [132,133]. An exception to this pattern has been observed in pancreatic cancer, where ESRP1 acts as a tumor suppressor while miR-23a, which directly targets the 3′UTR of *ESRP1*, promotes invasion and metastasis of cancer cells [71].

Considering these examples, miRNA-based regulation has clear implications involving RBM proteins and their alternative RNA splicing activities.

### 2.4. Indirect Modulators of miRNA-Based RNA Alternative Splicing Events

This review has mainly focused on the canonical mechanism of miRNA-based regulation, where a given miRNA directly targets a 3′UTR of an RNA splicing factor, dosing its expression, and leading to alternative RNA splicing events to target genes. Recently, several exciting new studies highlighted a few additional mechanisms, where the change in dosage of a given RNA splicing factor, is either due to the depletion of its target miRNAs through RNA sponges, or the depletion of transcription factors involved in transcribing the RNA splicing factors by miRNAs.

#### 2.4.1. circRNAs

Circular RNAs (circRNAs) are a recently discovered class of long ncRNAs in which the 5′ and 3′ termini are covalently linked, forming a circular structure. CircRNA are broadly expressed in mammalian cells and are involved in many cellular processes [134]. CircRNAs have been recently described as robust modulators of miRNA regulation of alternative RNA splicing factors, by acting as miRNA sponges. As expected, circRNA misregulation is correlated with specific forms of cancer, for example, ovarian cancer. A 2020 study identified that pervasive overexpression of ESRP1 in ovarian cancer is dependent on the expression of circ-0005585, which acts as an miRNA sponge for the ESRP1-specific miRNAs miR-15a/15b/16 and miR-23a/b, by expressing mutual miRNA targets as the 3′UTR of *ESRP1* [72]. The level of competition between circ-0005585 and the 3′UTR of *ESRP1* for mutual miRNAs was significant enough to allow ESRP1 overexpression, which suggest that circRNAs have a considerable influence on development and disease by modulating miRNA regulation.

In addition, it is important to note that circRNAs may modulate miRNA activity upstream of specific alternative RNA splicing factors, which nonetheless may significantly alter the downstream expression of a myriad of genes. This potent form of miRNA regulation is demonstrated by a 2018 study, which identified circANKS1B, a circRNA, to be distinctly correlated with breast cancer metastasis. Specifically, circANKS1B upregulated the expression of an ESRP1-specific transcription factor, USF1, by competitively binding the *USF1* 3′UTR-specific miRNAs. Thus, the increased expression of USF1 allowed for the increased expression of ESRP1 [135].

#### 2.4.2. Transcription Factors

CircRNAs are not the only modulators of miRNA-induced RNA alternative splicing events. Recently, an interesting study linked miRNA-controlled transcription factors with RNA alternative splicing events. The sequencing of RNAs in human airway epithelial cells undergoing EMT showed that miR-133a is significantly overexpressed in these cells [136], forcing these cells into spontaneous EMT, through the downregulation of the *grainyhead-like 2* (GRHL2) gene, an epithelial transcription factor involved in several disorders, such as the ectodermal dysplasia/short stature syndrome [137] and deafness autosomal dominant 28 [138]. The loss of GRHL2 expression led to the downregulation of the ESRP1 [136]. One of its characterized targets is *p120-catenin*. Loss of ESRP1 lead to an isoform switch that caused the loss of E-cadherin, which is one of the hallmarks of mesenchymal cells [136]. Although this is only one example, it is relatively common for transcription factors to possess many predicted and validated miRNA targets in their 3′UTRs, suggesting that perhaps this novel regulatory mechanism is more widespread.

## 3. Conclusions

It is clear that miRNAs are important modulators of alternative RNA splicing networks. Disruption of specific miRNA targeting pathways leads to RNA splicing errors that have been described in many types of cancers, neurological disorders, and developmental dysregulation. Unfortunately, apart from some of the examples discussed in this review, relatively little is known about splicing changes associated with micro-RNA-mediated repression. Still, the field is growing, with more interactions identified in recent years. In this review, we covered several examples of alternative RNA splicing induced by the dysregulation of RNA splicing factors. It is very challenging to pinpoint the direct effects that miRNAs have on alternative RNA splicing, but the widespread consequences of these changes contribute significantly to disease and developmental disorders.

## Figures and Tables

**Table 1:** Summary of the RNA splicing factors discussed in this review.

		miRNA(s)	Spliced Genes Affected	Development/Disease	Accession #(s)
**SR Proteins**	SRSF1	miR-10-a/b miR-28 miR-505 miR-766-3p	*Tau, Ich-1, Bcl-x*	Various cancers	[24,25,26,27,28]
	SRSF2	miR-183-5p miR-193a-3p miR-200c-3p	-	Various cancers	[28,29,30,31,32,33]
	SRSF6	miR-146a miR-193a-5p miR-506-3p	*OGDHL, ECM1*	Pancreatic cancer, Alzheimer’s disease, pleural fibrosis	[34,35,36,37]
	SRSF7	miR-30a-5p miR-181a-5p miR-188 miR-216b-5p	*SPP1*	Renal cancer, kidney injuries	[38,39]
	SRSF9	miR-1/206 miR-802	⎯	Bladder and cervical cancer	[40,41,42]
**hnRNPs**	hnRNP A1	miR-1-3p miR-135a-5p miR-137 miR-149-5p miR-206 miR-424 miR-451 miR-503	-	Acute myeloid leukemia, colon cancer	[28,43]
	hnRNP A2	miR-124 miR-340	*PKM*	-	[28]
**PTBs**	PTBP1	miR-1/206 miR-124 miR-133b miR-137 miR-194-5p miR-340	*PKM, APP*	Various cancers	[44,45,46,47]
	PTBP2	miR-132 miR-133	*Tau, PKM*	Glioblastoma	[48,49,50]
**RBFOX**	RBFOX1	miR-129-5p miR-980	-	-	[51,52,53]
	RBFOX2	let-7g miR-9 miR-135a	*Scn5a, Kcnd3*	Myotonic dystrophy type 1 (DM1)	[54]
	RBFOX3	miR-129-5p	-	-	[53]
**Quaking**	QKI-5	miR-143-3p miR-200c miR-214 miR-221 miR-375	-	Various cancers	[55,56,57,58]
	QKI-6	miR-29a miR-214	-	Glioblastoma	[59,60]
	QKI-7	miR-214	-	-	[59]
**CELF**	CELF1	miR-23a/b miR-322 miR-503	-	-	[23,61]
	CELF2	miR-20a miR-23a/b miR-95-3p	-	Glioblastoma	[23,62,63]
**NOVA**	NOVA1	miR-27a-3p miR-181b-5p miR-203a-3p miR-339 miR-592 miR-146b-5p	-	Gastric and thyroid cancers	[64,65,66,67]
	NOVA2	miR-7-5p	-	Non-small cell lung cancer	[68]
**RBM**	RBM10	miR-133a/b miR-335	*Numb*	Endometrial cancer	[69]
	RBM24	miR-222	*Coro6, Fxr1, NACA*	Myogenic differentiation	[70]
	ESRP1	miR-15a/b miR-16 miR-23a/b	*CD44, FGFR2, EPB41L5, Rac1*	Various cancers	[71,72]

## References

[1] Kim E., Magen A., Ast G. (2007). Different levels of alternative splicing among eukaryotes. Nucleic Acids Res..

[2] Han J., Ding J.H., Byeon C.W., Kim J.H., Hertel K.J., Jeong S., Fu X.D. (2011). SR proteins induce alternative exon skipping through their activities on the flanking constitutive exons. Mol. Cell Biol..

[3] Graveley B.R. (2000). Sorting out the complexity of SR protein functions. RNA.

[4] Matlin A.J., Clark F., Smith C.W. (2005). Understanding alternative splicing: Towards a cellular code. Nat. Rev. Mol. Cell Biol..

[5] Busch A., Hertel K.J. (2012). Evolution of SR protein and hnRNP splicing regulatory factors. Wiley Interdiscip. Rev. RNA.

[6] Long J.C., Caceres J.F. (2009). The SR protein family of splicing factors: Master regulators of gene expression. Biochem. J..

[7] Geuens T., Bouhy D., Timmerman V. (2016). The hnRNP family: Insights into their role in health and disease. Hum. Genet..

[8] Han S.P., Tang Y.H., Smith R. (2010). Functional diversity of the hnRNPs: Past, present and perspectives. Biochem. J..

[9] Gabut M., Chaudhry S., Blencowe B.J. (2008). SnapShot: The splicing regulatory machinery. Cell.

[10] Wei L., Lee S., Majumdar S., Zhang B., Sanfilippo P., Joseph B., Miura P., Soller M., Lai E.C. (2020). Overlapping Activities of ELAV/Hu Family RNA Binding Proteins Specify the Extended Neuronal 3′ UTR Landscape in Drosophila. Mol. Cell.

[11] Bartel D.P. (2018). Metazoan MicroRNAs. Cell.

[12] Wolter J.M., Le H.H., Linse A., Godlove V.A., Nguyen T.D., Kotagama K., Lynch A., Rawls A., Mangone M. (2017). Evolutionary patterns of metazoan microRNAs reveal targeting principles in the let-7 and miR-10 families. Genome Res..

[13] Cevec M., Thibaudeau C., Plavec J. (2010). NMR structure of the let-7 miRNA interacting with the site LCS1 of lin-41 mRNA from Caenorhabditis elegans. Nucleic Acids Res..

[14] Chen K., Rajewsky N. (2007). The evolution of gene regulation by transcription factors and microRNAs. Nat. Rev. Genet..

[15] Lee R.C., Feinbaum R.L., Ambros V. (1993). The C. elegans heterochronic gene lin-4 encodes small RNAs with antisense complementarity to lin-14. Cell.

[16] Reinhart B.J., Slack F.J., Basson M., Pasquinelli A.E., Bettinger J.C., Rougvie A.E., Horvitz H.R., Ruvkun G. (2000). The 21-nucleotide let-7 RNA regulates developmental timing in Caenorhabditis elegans. Nature.

[17] Pasquinelli A.E., Reinhart B.J., Slack F., Martindale M.Q., Kuroda M.I., Maller B., Hayward D.C., Ball E.E., Degnan B., Muller P. (2000). Conservation of the sequence and temporal expression of let-7 heterochronic regulatory RNA. Nature.

[18] Grimson A., Srivastava M., Fahey B., Woodcroft B.J., Chiang H.R., King N., Degnan B.M., Rokhsar D.S., Bartel D.P. (2008). Early origins and evolution of microRNAs and Piwi-interacting RNAs in animals. Nature.

[19] Kotagama K., Schorr A.L., Steber H.S., Mangone M. (2019). ALG-1 Influences Accurate mRNA Splicing Patterns in the Caenorhabditis elegans Intestine and Body Muscle Tissues by Modulating Splicing Factor Activities. Genetics.

[20] Landgraf P., Rusu M., Sheridan R., Sewer A., Iovino N., Aravin A., Pfeffer S., Rice A., Kamphorst A.O., Landthaler M. (2007). A mammalian microRNA expression atlas based on small RNA library sequencing. Cell.

[21] Eisenberg I., Eran A., Nishino I., Moggio M., Lamperti C., Amato A.A., Lidov H.G., Kang P.B., North K.N., Mitrani-Rosenbaum S. (2007). Distinctive patterns of microRNA expression in primary muscular disorders. Proc. Natl. Acad. Sci. USA.

[22] Jima D.D., Zhang J., Jacobs C., Richards K.L., Dunphy C.H., Choi W.W., Au W.Y., Srivastava G., Czader M.B., Rizzieri D.A. (2010). Deep sequencing of the small RNA transcriptome of normal and malignant human B cells identifies hundreds of novel microRNAs. Blood.

[23] Kalsotra A., Wang K., Li P.F., Cooper T.A. (2010). MicroRNAs coordinate an alternative splicing network during mouse postnatal heart development. Genes Dev..

[24] Verduci L., Simili M., Rizzo M., Mercatanti A., Evangelista M., Mariani L., Rainaldi G., Pitto L. (2010). MicroRNA (miRNA)-mediated interaction between leukemia/lymphoma-related factor (LRF) and alternative splicing factor/splicing factor 2 (ASF/SF2) affects mouse embryonic fibroblast senescence and apoptosis. J. Biol. Chem..

[25] Meseguer S., Mudduluru G., Escamilla J.M., Allgayer H., Barettino D. (2011). MicroRNAs-10a and -10b contribute to retinoic acid-induced differentiation of neuroblastoma cells and target the alternative splicing regulatory factor SFRS1 (SF2/ASF). J. Biol. Chem..

[26] Anczukow O., Akerman M., Clery A., Wu J., Shen C., Shirole N.H., Raimer A., Sun S., Jensen M.A., Hua Y. (2015). SRSF1-Regulated Alternative Splicing in Breast Cancer. Mol. Cell.

[27] Chen C., Xue S., Zhang J., Chen W., Gong D., Zheng J., Ma J., Xue W., Chen Y., Zhai W. (2017). DNA-methylation-mediated repression of miR-766-3p promotes cell proliferation via targeting SF2 expression in renal cell carcinoma. Int. J. Cancer.

[28] Sokol E., Kedzierska H., Czubaty A., Rybicka B., Rodzik K., Tanski Z., Boguslawska J., Piekielko-Witkowska A. (2018). microRNA-mediated regulation of splicing factors SRSF1, SRSF2 and hnRNP A1 in context of their alternatively spliced 3′UTRs. Exp. Cell Res..

[29] Luo C., Cheng Y., Liu Y., Chen L., Liu L., Wei N., Xie Z., Wu W., Feng Y. (2017). SRSF2 Regulates Alternative Splicing to Drive Hepatocellular Carcinoma Development. Cancer Res..

[30] Liang Y., Tebaldi T., Rejeski K., Joshi P., Stefani G., Taylor A., Song Y., Vasic R., Maziarz J., Balasubramanian K. (2018). SRSF2 mutations drive oncogenesis by activating a global program of aberrant alternative splicing in hematopoietic cells. Leukemia.

[31] Merdzhanova G., Edmond V., De Seranno S., Van den Broeck A., Corcos L., Brambilla C., Brambilla E., Gazzeri S., Eymin B. (2008). E2F1 controls alternative splicing pattern of genes involved in apoptosis through upregulation of the splicing factor SC35. Cell Death Differ..

[32] Kedzierska H., Poplawski P., Hoser G., Rybicka B., Rodzik K., Sokol E., Boguslawska J., Tanski Z., Fogtman A., Koblowska M. (2016). Decreased Expression of SRSF2 Splicing Factor Inhibits Apoptotic Pathways in Renal Cancer. Int. J. Mol. Sci..

[33] Kong L., Wei Q., Hu X., Chen L., Li J. (2019). miR-193a-3p Promotes Radio-Resistance of Nasopharyngeal Cancer Cells by Targeting SRSF2 Gene and Hypoxia Signaling Pathway. Med. Sci. Monit. Basic Res..

[34] Li M., Wu P., Yang Z., Deng S., Ni L., Zhang Y., Jin L., Pan Y. (2020). miR-193a-5p promotes pancreatic cancer cell metastasis through SRSF6-mediated alternative splicing of OGDHL and ECM1. Am. J. Cancer Res..

[35] Fernandez-Nogales M., Cabrera J.R., Santos-Galindo M., Hoozemans J.J., Ferrer I., Rozemuller A.J., Hernandez F., Avila J., Lucas J.J. (2014). Huntington’s disease is a four-repeat tauopathy with tau nuclear rods. Nat. Med..

[36] Mai H., Fan W., Wang Y., Cai Y., Li X., Chen F., Chen X., Yang J., Tang P., Chen H. (2019). Intranasal Administration of miR-146a Agomir Rescued the Pathological Process and Cognitive Impairment in an AD Mouse Model. Mol. Nucleic Acids.

[37] Liang L.M., Xiong L., Cheng P.P., Chen S.J., Feng X., Zhou Y.Y., Niu Q., Wang M., Chen Q., Song L.J. (2021). Splicing factor SRSF6 mediates pleural fibrosis. JCI Insight.

[38] Boguslawska J., Sokol E., Rybicka B., Czubaty A., Rodzik K., Piekielko-Witkowska A. (2016). microRNAs target SRSF7 splicing factor to modulate the expression of osteopontin splice variants in renal cancer cells. Gene.

[39] Liu B., Chai Y., Guo W., Lin K., Chen S., Liu J., Sun G., Chen G., Song F., He Y. (2019). MicroRNA-188 aggravates contrast-induced apoptosis by targeting SRSF7 in novel isotonic contrast-induced acute kidney injury rat models and renal tubular epithelial cells. Ann. Transl. Med..

[40] Bjorkman K.K., Buvoli M., Pugach E.K., Polmear M.M., Leinwand L.A. (2019). miR-1/206 downregulates splicing factor Srsf9 to promote C2C12 differentiation. Skelet Muscle.

[41] Yoshino H., Enokida H., Chiyomaru T., Tatarano S., Hidaka H., Yamasaki T., Gotannda T., Tachiwada T., Nohata N., Yamane T. (2012). Tumor suppressive microRNA-1 mediated novel apoptosis pathways through direct inhibition of splicing factor serine/arginine-rich 9 (SRSF9/SRp30c) in bladder cancer. Biochem. Biophys. Res. Commun..

[42] Zhang Q., Lv R., Guo W., Li X. (2019). microRNA-802 inhibits cell proliferation and induces apoptosis in human cervical cancer by targeting serine/arginine-rich splicing factor 9. J. Cell Biochem..

[43] Song L., Lin H.S., Gong J.N., Han H., Wang X.S., Su R., Chen M.T., Shen C., Ma Y.N., Yu J. (2017). microRNA-451-modulated hnRNP A1 takes a part in granulocytic differentiation regulation and acute myeloid leukemia. Oncotarget.

[44] Makeyev E.V., Zhang J., Carrasco M.A., Maniatis T. (2007). The MicroRNA miR-124 promotes neuronal differentiation by triggering brain-specific alternative pre-mRNA splicing. Mol. Cell.

[45] Taniguchi K., Sugito N., Shinohara H., Kuranaga Y., Inomata Y., Komura K., Uchiyama K., Akao Y. (2018). Organ-Specific MicroRNAs (MIR122, 137, and 206) Contribute to Tissue Characteristics and Carcinogenesis by Regulating Pyruvate Kinase M1/2 (PKM) Expression. Int. J. Mol. Sci..

[46] Smith P., Al Hashimi A., Girard J., Delay C., Hebert S.S. (2011). In vivo regulation of amyloid precursor protein neuronal splicing by microRNAs. J. Neurochem..

[47] Taniguchi K., Sakai M., Sugito N., Kumazaki M., Shinohara H., Yamada N., Nakayama T., Ueda H., Nakagawa Y., Ito Y. (2016). PTBP1-associated microRNA-1 and -133b suppress the Warburg effect in colorectal tumors. Oncotarget.

[48] Boutz P.L., Chawla G., Stoilov P., Black D.L. (2007). MicroRNAs regulate the expression of the alternative splicing factor nPTB during muscle development. Genes Dev..

[49] Lou S., Ji J., Cheng X., Ruan J., Li R., Guo Z. (2017). Oncogenic miR132 sustains proliferation and selfrenewal potential by inhibition of polypyrimidine tractbinding protein 2 in glioblastoma cells. Mol. Med. Rep..

[50] Smith P.Y., Delay C., Girard J., Papon M.A., Planel E., Sergeant N., Buee L., Hebert S.S. (2011). MicroRNA-132 loss is associated with tau exon 10 inclusion in progressive supranuclear palsy. Hum. Mol. Genet..

[51] Guven-Ozkan T., Busto G.U., Schutte S.S., Cervantes-Sandoval I., O’Dowd D.K., Davis R.L. (2016). MiR-980 Is a Memory Suppressor MicroRNA that Regulates the Autism-Susceptibility Gene A2bp1. Cell Rep..

[52] Kucherenko M.M., Shcherbata H.R. (2018). Stress-dependent miR-980 regulation of Rbfox1/A2bp1 promotes ribonucleoprotein granule formation and cell survival. Nat. Commun..

[53] Rajman M., Metge F., Fiore R., Khudayberdiev S., Aksoy-Aksel A., Bicker S., Ruedell Reschke C., Raoof R., Brennan G.P., Delanty N. (2017). A microRNA-129-5p/Rbfox crosstalk coordinates homeostatic downscaling of excitatory synapses. EMBO J..

[54] Misra C., Bangru S., Lin F., Lam K., Koenig S.N., Lubbers E.R., Hedhli J., Murphy N.P., Parker D.J., Dobrucki L.W. (2020). Aberrant Expression of a Non-muscle RBFOX2 Isoform Triggers Cardiac Conduction Defects in Myotonic Dystrophy. Dev. Cell.

[55] Caines R., Cochrane A., Kelaini S., Vila-Gonzalez M., Yang C., Eleftheriadou M., Moez A., Stitt A.W., Zeng L., Grieve D.J. (2019). The RNA-binding protein QKI controls alternative splicing in vascular cells, producing an effective model for therapy. J. Cell Sci..

[56] Larocque D., Fragoso G., Huang J., Mushynski W.E., Loignon M., Richard S., Almazan G. (2009). The QKI-6 and QKI-7 RNA binding proteins block proliferation and promote Schwann cell myelination. PLoS ONE.

[57] Kondo T., Furuta T., Mitsunaga K., Ebersole T.A., Shichiri M., Wu J., Artzt K., Yamamura K., Abe K. (1999). Genomic organization and expression analysis of the mouse qkI locus. Mamm. Genome.

[58] Wu J.I., Reed R.B., Grabowski P.J., Artzt K. (2002). Function of quaking in myelination: Regulation of alternative splicing. Proc. Natl. Acad. Sci. USA.

[59] Irie K., Tsujimura K., Nakashima H., Nakashima K. (2016). MicroRNA-214 Promotes Dendritic Development by Targeting the Schizophrenia-associated Gene Quaking (Qki). J. Biol. Chem..

[60] Xi Z., Wang P., Xue Y., Shang C., Liu X., Ma J., Li Z., Li Z., Bao M., Liu Y. (2017). Overexpression of miR-29a reduces the oncogenic properties of glioblastoma stem cells by downregulating Quaking gene isoform 6. Oncotarget.

[61] Shen X., Soibam B., Benham A., Xu X., Chopra M., Peng X., Yu W., Bao W., Liang R., Azares A. (2016). miR-322/-503 cluster is expressed in the earliest cardiac progenitor cells and drives cardiomyocyte specification. Proc. Natl. Acad. Sci. USA.

[62] Fan B., Jiao B.H., Fan F.S., Lu S.K., Song J., Guo C.Y., Yang J.K., Yang L. (2015). Downregulation of miR-95-3p inhibits proliferation, and invasion promoting apoptosis of glioma cells by targeting CELF2. Int. J. Oncol..

[63] Liao C., Chen W., Wang J. (2019). MicroRNA-20a Regulates Glioma Cell Proliferation, Invasion, and Apoptosis by Targeting CUGBP Elav-Like Family Member 2. World Neurosurg..

[64] Zhi F., Wang Q., Deng D., Shao N., Wang R., Xue L., Wang S., Xia X., Yang Y. (2014). MiR-181b-5p downregulates NOVA1 to suppress proliferation, migration and invasion and promote apoptosis in astrocytoma. PLoS ONE.

[65] Herve M., Ibrahim E.C. (2016). MicroRNA screening identifies a link between NOVA1 expression and a low level of IKAP in familial dysautonomia. Dis. Model. Mech..

[66] Luo Y., Hao T., Zhang J., Zhang M., Sun P., Wu L. (2019). MicroRNA-592 suppresses the malignant phenotypes of thyroid cancer by regulating lncRNA NEAT1 and downregulating NOVA1. Int. J. Mol. Med..

[67] Shen B., Zhang Y., Yu S., Yuan Y., Zhong Y., Lu J., Feng J. (2015). MicroRNA-339, an epigenetic modulating target is involved in human gastric carcinogenesis through targeting NOVA1. FEBS Lett..

[68] Xiao H. (2019). MiR-7-5p suppresses tumor metastasis of non-small cell lung cancer by targeting NOVA2. Cell Mol. Biol. Lett..

[69] Dou X.Q., Chen X.J., Zhou Q., Wen M.X., Zhang S.Z., Zhang S.Q. (2020). miR-335 modulates Numb alternative splicing via targeting RBM10 in endometrial cancer. Kaohsiung J. Med. Sci..

[70] Cardinali B., Cappella M., Provenzano C., Garcia-Manteiga J.M., Lazarevic D., Cittaro D., Martelli F., Falcone G. (2016). MicroRNA-222 regulates muscle alternative splicing through Rbm24 during differentiation of skeletal muscle cells. Cell Death Dis..

[71] Wu G., Li Z., Jiang P., Zhang X., Xu Y., Chen K., Li X. (2017). MicroRNA-23a promotes pancreatic cancer metastasis by targeting epithelial splicing regulator protein 1. Oncotarget.

[72] Deng G., Zhou X., Chen L., Yao Y., Li J., Zhang Y., Luo C., Sun L., Tang J. (2020). High expression of ESRP1 regulated by circ-0005585 promotes cell colonization in ovarian cancer. Cancer Cell Int..

[73] Manley J.L., Krainer A.R. (2010). A rational nomenclature for serine/arginine-rich protein splicing factors (SR proteins). Genes Dev..

[74] Fischer D.C., Noack K., Runnebaum I.B., Watermann D.O., Kieback D.G., Stamm S., Stickeler E. (2004). Expression of splicing factors in human ovarian cancer. Oncol. Rep..

[75] Ghigna C., Moroni M., Porta C., Riva S., Biamonti G. (1998). Altered expression of heterogenous nuclear ribonucleoproteins and SR factors in human colon adenocarcinomas. Cancer Res..

[76] Paronetto M.P., Achsel T., Massiello A., Chalfant C.E., Sette C. (2007). The RNA-binding protein Sam68 modulates the alternative splicing of Bcl-x. J. Cell Biol..

[77] Voigt A., Zintl F. (2003). Effects of retinoic acid on proliferation, apoptosis, cytotoxicity, migration, and invasion of neuroblastoma cells. Med. Pediatr. Oncol..

[78] Karni R., de Stanchina E., Lowe S.W., Sinha R., Mu D., Krainer A.R. (2007). The gene encoding the splicing factor SF2/ASF is a proto-oncogene. Nat. Struct. Mol. Biol..

[79] Ghigna C., Giordano S., Shen H., Benvenuto F., Castiglioni F., Comoglio P.M., Green M.R., Riva S., Biamonti G. (2005). Cell motility is controlled by SF2/ASF through alternative splicing of the Ron protooncogene. Mol. Cell.

[80] Anczukow O., Rosenberg A.Z., Akerman M., Das S., Zhan L., Karni R., Muthuswamy S.K., Krainer A.R. (2012). The splicing factor SRSF1 regulates apoptosis and proliferation to promote mammary epithelial cell transformation. Nat. Struct. Mol. Biol..

[81] Goncalves V., Jordan P. (2015). Posttranscriptional Regulation of Splicing Factor SRSF1 and Its Role in Cancer Cell Biology. Biomed. Res. Int..

[82] You Y., Que K., Zhou Y., Zhang Z., Zhao X., Gong J., Liu Z. (2018). MicroRNA-766-3p Inhibits Tumour Progression by Targeting Wnt3a in Hepatocellular Carcinoma. Mol. Cells.

[83] Lan G., Xie W., Li L., Zhang M., Liu D., Tan Y.L., Cheng H.P., Gong D., Huang C., Zheng X.L. (2016). MicroRNA-134 actives lipoprotein lipase-mediated lipid accumulation and inflammatory response by targeting angiopoietin-like 4 in THP-1 macrophages. Biochem. Biophys. Res. Commun..

[84] Li K., Wang Z. (2021). Splicing factor SRSF2-centric gene regulation. Int. J. Biol. Sci..

[85] Cohen-Eliav M., Golan-Gerstl R., Siegfried Z., Andersen C.L., Thorsen K., Orntoft T.F., Mu D., Karni R. (2013). The splicing factor SRSF6 is amplified and is an oncoprotein in lung and colon cancers. J. Pathol..

[86] Yin X., Jin N., Gu J., Shi J., Zhou J., Gong C.X., Iqbal K., Grundke-Iqbal I., Liu F. (2012). Dual-specificity tyrosine phosphorylation-regulated kinase 1A (Dyrk1A) modulates serine/arginine-rich protein 55 (SRp55)-promoted Tau exon 10 inclusion. J. Biol. Chem..

[87] Raponi M., Kralovicova J., Copson E., Divina P., Eccles D., Johnson P., Baralle D., Vorechovsky I. (2011). Prediction of single-nucleotide substitutions that result in exon skipping: Identification of a splicing silencer in BRCA1 exon 6. Hum. Mutat..

[88] Cloutier P., Toutant J., Shkreta L., Goekjian S., Revil T., Chabot B. (2008). Antagonistic effects of the SRp30c protein and cryptic 5′ splice sites on the alternative splicing of the apoptotic regulator Bcl-x. J. Biol. Chem..

[89] Clarke J.P., Thibault P.A., Salapa H.E., Levin M.C. (2021). A Comprehensive Analysis of the Role of hnRNP A1 Function and Dysfunction in the Pathogenesis of Neurodegenerative Disease. Front. Mol. Biosci..

[90] Han K., Yeo G., An P., Burge C.B., Grabowski P.J. (2005). A combinatorial code for splicing silencing: UAGG and GGGG motifs. PLoS Biol..

[91] Otsuka K., Yamamoto Y., Ochiya T. (2018). Regulatory role of resveratrol, a microRNA-controlling compound, in HNRNPA1 expression, which is associated with poor prognosis in breast cancer. Oncotarget.

[92] Biamonti G., Ruggiu M., Saccone S., Della Valle G., Riva S. (1994). Two homologous genes, originated by duplication, encode the human hnRNP proteins A2 and A1. Nucleic Acids Res..

[93] Clower C.V., Chatterjee D., Wang Z., Cantley L.C., Vander Heiden M.G., Krainer A.R. (2010). The alternative splicing repressors hnRNP A1/A2 and PTB influence pyruvate kinase isoform expression and cell metabolism. Proc. Natl. Acad. Sci. USA.

[94] Sun Y., Zhao X., Zhou Y., Hu Y. (2012). miR-124, miR-137 and miR-340 regulate colorectal cancer growth via inhibition of the Warburg effect. Oncol. Rep..

[95] Chen M., Zhang J., Manley J.L. (2010). Turning on a fuel switch of cancer: hnRNP proteins regulate alternative splicing of pyruvate kinase mRNA. Cancer Res..

[96] Moran-Jones K., Grindlay J., Jones M., Smith R., Norman J.C. (2009). hnRNP A2 regulates alternative mRNA splicing of TP53INP2 to control invasive cell migration. Cancer Res..

[97] Konno M., Koseki J., Kawamoto K., Nishida N., Matsui H., Dewi D.L., Ozaki M., Noguchi Y., Mimori K., Gotoh N. (2015). Embryonic MicroRNA-369 Controls Metabolic Splicing Factors and Urges Cellular Reprograming. PLoS ONE.

[98] Vasudevan S., Tong Y., Steitz J.A. (2007). Switching from repression to activation: microRNAs can up-regulate translation. Science.

[99] Sawicka K., Bushell M., Spriggs K.A., Willis A.E. (2008). Polypyrimidine-tract-binding protein: A multifunctional RNA-binding protein. Biochem. Soc. Trans..

[100] Wu H., Sun S., Tu K., Gao Y., Xie B., Krainer A.R., Zhu J. (2010). A splicing-independent function of SF2/ASF in microRNA processing. Mol. Cell.

[101] Xue Y., Ouyang K., Huang J., Zhou Y., Ouyang H., Li H., Wang G., Wu Q., Wei C., Bi Y. (2013). Direct conversion of fibroblasts to neurons by reprogramming PTB-regulated microRNA circuits. Cell.

[102] Xue Y., Qian H., Hu J., Zhou B., Zhou Y., Hu X., Karakhanyan A., Pang Z., Fu X.D. (2016). Sequential regulatory loops as key gatekeepers for neuronal reprogramming in human cells. Nat. Neurosci..

[103] Takahashi H., Nishimura J., Kagawa Y., Kano Y., Takahashi Y., Wu X., Hiraki M., Hamabe A., Konno M., Haraguchi N. (2015). Significance of Polypyrimidine Tract-Binding Protein 1 Expression in Colorectal Cancer. Mol. Cancer.

[104] Silber J., Lim D.A., Petritsch C., Persson A.I., Maunakea A.K., Yu M., Vandenberg S.R., Ginzinger D.G., James C.D., Costello J.F. (2008). miR-124 and miR-137 inhibit proliferation of glioblastoma multiforme cells and induce differentiation of brain tumor stem cells. BMC Med..

[105] Taniguchi K., Ito Y., Sugito N., Kumazaki M., Shinohara H., Yamada N., Nakagawa Y., Sugiyama T., Futamura M., Otsuki Y. (2015). Organ-specific PTB1-associated microRNAs determine expression of pyruvate kinase isoforms. Sci. Rep..

[106] Christofk H.R., Vander Heiden M.G., Harris M.H., Ramanathan A., Gerszten R.E., Wei R., Fleming M.D., Schreiber S.L., Cantley L.C. (2008). The M2 splice isoform of pyruvate kinase is important for cancer metabolism and tumour growth. Nature.

[107] Missiaglia E., Shepherd C.J., Patel S., Thway K., Pierron G., Pritchard-Jones K., Renard M., Sciot R., Rao P., Oberlin O. (2010). MicroRNA-206 expression levels correlate with clinical behaviour of rhabdomyosarcomas. Br. J. Cancer.

[108] Conboy J.G. (2017). Developmental regulation of RNA processing by Rbfox proteins. Wiley Interdiscip. Rev. RNA.

[109] Skipper M., Milne C.A., Hodgkin J. (1999). Genetic and molecular analysis of fox-1, a numerator element involved in Caenorhabditis elegans primary sex determination. Genetics.

[110] Jin Y., Suzuki H., Maegawa S., Endo H., Sugano S., Hashimoto K., Yasuda K., Inoue K. (2003). A vertebrate RNA-binding protein Fox-1 regulates tissue-specific splicing via the pentanucleotide GCAUG. EMBO J..

[111] Fogel B.L., Wexler E., Wahnich A., Friedrich T., Vijayendran C., Gao F., Parikshak N., Konopka G., Geschwind D.H. (2012). RBFOX1 regulates both splicing and transcriptional networks in human neuronal development. Hum. Mol. Genet..

[112] Martin C.L., Duvall J.A., Ilkin Y., Simon J.S., Arreaza M.G., Wilkes K., Alvarez-Retuerto A., Whichello A., Powell C.M., Rao K. (2007). Cytogenetic and molecular characterization of A2BP1/FOX1 as a candidate gene for autism. Am. J. Med. Genet. B Neuropsychiatr. Genet..

[113] Tian B., Manley J.L. (2013). Alternative cleavage and polyadenylation: The long and short of it. Trends Biochem. Sci..

[114] Boutet S.C., Cheung T.H., Quach N.L., Liu L., Prescott S.L., Edalati A., Iori K., Rando T.A. (2012). Alternative polyadenylation mediates microRNA regulation of muscle stem cell function. Cell Stem Cell.

[115] Blazie S.M., Geissel H.C., Wilky H., Joshi R., Newbern J., Mangone M. (2017). Alternative Polyadenylation Directs Tissue-Specific miRNA Targeting in Caenorhabditis elegans Somatic Tissues. Genetics.

[116] Freyermuth F., Rau F., Kokunai Y., Linke T., Sellier C., Nakamori M., Kino Y., Arandel L., Jollet A., Thibault C. (2016). Splicing misregulation of SCN5A contributes to cardiac-conduction delay and heart arrhythmia in myotonic dystrophy. Nat. Commun..

[117] Dasgupta T., Ladd A.N. (2012). The importance of CELF control: Molecular and biological roles of the CUG-BP, Elav-like family of RNA-binding proteins. Wiley Interdiscip. Rev. RNA.

[118] Yoon S.O., Kim E.K., Lee M., Jung W.Y., Lee H., Kang Y., Jang Y.J., Hong S.W., Choi S.H., Yang W.I. (2016). NOVA1 inhibition by miR-146b-5p in the remnant tissue microenvironment defines occult residual disease after gastric cancer removal. Oncotarget.

[119] Li K., Zhu X., Chen X., Wang X. (2020). MicroRNA27a3p promotes epithelialmesenchymal transition by targeting NOVA alternative splicing regulator 1 in gastric cancer. Mol. Med. Rep..

[120] Meldolesi J. (2020). Alternative Splicing by NOVA Factors: From Gene Expression to Cell Physiology and Pathology. Int. J. Mol. Sci..

[121] Kim E.K., Yoon S.O., Jung W.Y., Lee H., Kang Y., Jang Y.J., Hong S.W., Choi S.H., Yang W.I. (2017). Implications of NOVA1 suppression within the microenvironment of gastric cancer: Association with immune cell dysregulation. Gastric Cancer.

[122] Ebersole T.A., Chen Q., Justice M.J., Artzt K. (1996). The quaking gene product necessary in embryogenesis and myelination combines features of RNA binding and signal transduction proteins. Nat. Genet..

[123] Galarneau A., Richard S. (2005). Target RNA motif and target mRNAs of the Quaking STAR protein. Nat. Struct. Mol. Biol..

[124] Shu P., Fu H., Zhao X., Wu C., Ruan X., Zeng Y., Liu W., Wang M., Hou L., Chen P. (2017). MicroRNA-214 modulates neural progenitor cell differentiation by targeting Quaking during cerebral cortex development. Sci. Rep..

[125] van Mil A., Grundmann S., Goumans M.J., Lei Z., Oerlemans M.I., Jaksani S., Doevendans P.A., Sluijter J.P. (2012). MicroRNA-214 inhibits angiogenesis by targeting Quaking and reducing angiogenic growth factor release. Cardiovasc. Res..

[126] Pillman K.A., Phillips C.A., Roslan S., Toubia J., Dredge B.K., Bert A.G., Lumb R., Neumann D.P., Li X., Conn S.J. (2018). miR-200/375 control epithelial plasticity-associated alternative splicing by repressing the RNA-binding protein Quaking. EMBO J..

[127] Mukohyama J., Isobe T., Hu Q., Hayashi T., Watanabe T., Maeda M., Yanagi H., Qian X., Yamashita K., Minami H. (2019). miR-221 Targets QKI to Enhance the Tumorigenic Capacity of Human Colorectal Cancer Stem Cells. Cancer Res..

[128] He Z., Yi J., Liu X., Chen J., Han S., Jin L., Chen L., Song H. (2016). MiR-143-3p functions as a tumor suppressor by regulating cell proliferation, invasion and epithelial-mesenchymal transition by targeting QKI-5 in esophageal squamous cell carcinoma. Mol. Cancer.

[129] Sutherland L.C., Rintala-Maki N.D., White R.D., Morin C.D. (2005). RNA binding motif (RBM) proteins: A novel family of apoptosis modulators?. J. Cell Biochem..

[130] Yang Y., Park J.W., Bebee T.W., Warzecha C.C., Guo Y., Shang X., Xing Y., Carstens R.P. (2016). Determination of a Comprehensive Alternative Splicing Regulatory Network and Combinatorial Regulation by Key Factors during the Epithelial-to-Mesenchymal Transition. Mol. Cell Biol..

[131] Ishii H., Saitoh M., Sakamoto K., Kondo T., Katoh R., Tanaka S., Motizuki M., Masuyama K., Miyazawa K. (2014). Epithelial splicing regulatory proteins 1 (ESRP1) and 2 (ESRP2) suppress cancer cell motility via different mechanisms. J. Biol. Chem..

[132] Warzecha C.C., Sato T.K., Nabet B., Hogenesch J.B., Carstens R.P. (2009). ESRP1 and ESRP2 are epithelial cell-type-specific regulators of FGFR2 splicing. Mol. Cell.

[133] Fagoonee S., Picco G., Orso F., Arrigoni A., Longo D.L., Forni M., Scarfo I., Cassenti A., Piva R., Cassoni P. (2017). The RNA-binding protein ESRP1 promotes human colorectal cancer progression. Oncotarget.

[134] Yu C.Y., Kuo H.C. (2019). The emerging roles and functions of circular RNAs and their generation. J. Biomed. Sci..

[135] Zeng K., He B., Yang B.B., Xu T., Chen X., Xu M., Liu X., Sun H., Pan Y., Wang S. (2018). The pro-metastasis effect of circANKS1B in breast cancer. Mol. Cancer.

[136] Chen L., He X., Xie Y., Huang Y., Wolff D.W., Abel P.W., Tu Y. (2018). Up-regulated miR-133a orchestrates epithelial-mesenchymal transition of airway epithelial cells. Sci. Rep..

[137] Walne A.J., Collopy L., Cardoso S., Ellison A., Plagnol V., Albayrak C., Albayrak D., Kilic S.S., Patiroglu T., Akar H. (2016). Marked overlap of four genetic syndromes with dyskeratosis congenita confounds clinical diagnosis. Haematologica.

[138] Vona B., Nanda I., Neuner C., Muller T., Haaf T. (2013). Confirmation of GRHL2 as the gene for the DFNA28 locus. Am. J. Med. Genet. A.

